# Development of an Efficient Protein Extraction Method Compatible with LC-MS/MS for Proteome Mapping in Two Australian Seagrasses *Zostera muelleri* and *Posidonia australis*

**DOI:** 10.3389/fpls.2017.01416

**Published:** 2017-08-15

**Authors:** Zhijian Jiang, Manoj Kumar, Matthew P. Padula, Mathieu Pernice, Tim Kahlke, Mikael Kim, Peter J. Ralph

**Affiliations:** ^1^Key Laboratory of Tropical Marine Bio-resources and Ecology, South China Sea Institute of Oceanology, Chinese Academy of Sciences Guangzhou, China; ^2^Climate Change Cluster (C3), Faculty of Science, University of Technology Sydney (UTS) Sydney, NSW, Australia; ^3^Proteomics Core Facility, University of Technology Sydney (UTS) Sydney, NSW, Australia

**Keywords:** seagrass, proteomics, 2D-IEF, *Zostera muelleri*, *Posidonia australis*, LC-MS/MS

## Abstract

The availability of the first complete genome sequence of the marine flowering plant *Zostera marina* (commonly known as seagrass) in early 2016, is expected to significantly raise the impact of seagrass proteomics. Seagrasses are marine ecosystem engineers that are currently declining worldwide at an alarming rate due to both natural and anthropogenic disturbances. Seagrasses (especially species of the genus *Zostera*) are compromised for proteomic studies primarily due to the lack of efficient protein extraction methods because of their recalcitrant cell wall which is rich in complex polysaccharides and a high abundance of secondary metabolites in their cells. In the present study, three protein extraction methods that are commonly used in plant proteomics i.e., phenol (P); trichloroacetic acid/acetone/SDS/phenol (TASP); and borax/polyvinyl-polypyrrolidone/phenol (BPP) extraction, were evaluated quantitatively and qualitatively based on two dimensional isoelectric focusing (2D-IEF) maps and LC-MS/MS analysis using the two most abundant Australian seagrass species, namely *Zostera muelleri* and *Posidonia australis*. All three tested methods produced high quality protein extracts with excellent 2D-IEF maps in *P. australis*. However, the BPP method produces better results in *Z. muelleri* compared to TASP and P. Therefore, we further modified the BPP method (M-BPP) by homogenizing the tissue in a modified protein extraction buffer containing both ionic and non-ionic detergents (0.5% SDS; 1.5% Triton X-100), 2% PVPP and protease inhibitors. Further, the extracted proteins were solubilized in 0.5% of zwitterionic detergent (C7BzO) instead of 4% CHAPS. This slight modification to the BPP method resulted in a higher protein yield, and good quality 2-DE maps with a higher number of protein spots in both the tested seagrasses. Further, the M-BPP method was successfully utilized in western-blot analysis of phosphoenolpyruvate carboxylase (PEPC—a key enzyme for carbon metabolism). This optimized protein extraction method will be a significant stride toward seagrass proteome mining and identifying the protein biomarkers to stress response of seagrasses under the scenario of global climate change and anthropogenic perturbations.

## Introduction

Seagrasses (marine flowering plants), are marine ecological engineers delivering a range of ecologically and economically valuable biological services to marine aquatic ecosystems (Larkum et al., 2006). They are rated the third most valuable ecosystem globally with the average global value for their ecological services estimated at US $28,916 ha^−1^ yr^−1^ (Costanza et al., 2014). However, seagrasses are currently facing a global crisis and are declining at an alarming rate (by >7% yr^−1^) due to both natural and anthropogenic disturbances (Waycott et al., 2009).

Understanding of acclimation and/or tolerance mechanism of seagrasses to external perturbations is highly critical for developing strategies to prevent the loss of seagrass meadow (Davey et al., 2016; Kumar et al., 2016). Toward this, identification of biomarkers such as protein markers has been suggested as a possible solution that can provide early warning signals to prevent the seagrass meadow's demise before they pass the point of no return (Macreadie et al., 2014). Since, proteins respond dynamically to environmental fluctuations, the proteomics can provide novel insights into cellular pathways and biochemistry. To understand the change in state of the proteins, it is now common to perform a differential display of the proteome under contrasting conditions. Proteomics using advanced mass-spectrometry based approaches have had an increasing impact on the study of terrestrial plant responses to (a) biotic stresses (see references in Kumar et al., 2017). However, proteomics in seagrasses is still in its incipient stage for two primary reasons—(1) the lack of efficient protein extraction methods, and (2) limited availability of genomic sequence information. However, the recently published genome sequence of *Zostera marina* (Olsen et al., 2016) enables seagrass researchers to integrate additional—omics data types such as genomics and transcriptomics into their analysis of the physiological and molecular responses to environmental stress. Therefore, investigations into the seagrass proteome are important since proteins, unlike mRNA, are the direct effectors of the plant stress response.

Two-dimensional polyacrylamide gel electrophoresis (2-DE), established by O'Farrell, coupled with mass spectrometry (MS), is a cost effective and widely used proteomic technique. However, relatively expensive, alternative gel-free proteomic approaches such as isotopic labeling (iTRAQ and TMT) and Data Independent Acquisition (DIA/SWATH) are rapidly emerging (Hu et al., 2015). Irrespective of the technique used for proteomic studies, effective protein extraction and solubilization are unquestionably the critical factors in obtaining comprehensive proteome analysis. The comprehensive, unbiased extraction of protein from marine plants is particularly challenging due to their recalcitrant cell wall and low protein content. Moreover, their cell wall and vacuoles that make majority of the cell mass are associated with several secondary metabolites that strongly interfere with 2-DE, resulting in horizontal and vertical streaking, smearing, and reduced numbers of distinctly resolved protein spots (Wu et al., 2014a). The most common interfering substances in seagrasses are phenolic compounds, proteolytic and oxidative enzymes, terpenes, pigments, organic acids, and complex cell wall polysaccharides such as lignin (Papenbrock, 2012).

For recalcitrant plant tissues, protein extraction methods are typically based on trichloroacetic acid (TCA)/acetone washing or precipitation steps followed by phenol extraction. Recently, Wu et al. (2014b) formulated a “universal protein extraction protocol” for plant tissue by integrating TCA/acetone and phenol based methods with SDS extraction buffer to provide an improved 2-DE based proteomic analysis for most of the terrestrial plant tissues. However, TCA/acetone- and phenol-based methods are lengthy and involve multiple washing steps, resulting in unavoidable loss of protein. A protein extraction protocol designed for halophytes includes chemicals such as borax, polyvinyl-polypyrrolidone, phenol (BPP) and triton X-100 in the extraction buffer, has been shown to be effective in removing interfering compounds and salts in a relatively shorter time without protein loss since it does not involve multiple washing steps (Wang et al., 2007). To date, no common and simple protocol exists for protein extraction that can be used on a large scale for marine plant proteomics, however few attempts have been undertaken previously to obtain well-resolved 1-DE and 2-DE images in seagrasses (Spadafora et al., 2008; Serra and Mazzuca, 2011 and references therein). There is a critical need for such a rapid and efficient protocol, especially for projects wherein comparative proteomic analysis is required for seagrass samples exposed naturally or in laboratory conditions to diverse a(biotic) stresses. Such protocols should also be effective for protein extraction for a range of marine plant species and also for different tissues.

In view of this and considering the fact that there are no reports on optimized protein extraction protocols for species of the genus *Zostera*, we compare three commonly used plant protein extraction methods (P, TASP, and BPP) for 2-DE separation. For this, the whole leaf tissue of two dominant seagrasses of Australia named *Zostera muelleri* and *Posidonia australis* were used as an experimental model.

## Materials and methods

### Plant material

Samples of *Z. muelleri* were harvested from Narrabeen Lagoon (New South Wales, Australia) while samples of *P. australis* were collected from Ports Stephens (New South Wales, Australia). Turfs of seagrass with 10–15 cm of intact sediment were carefully removed from the meadow using a hand spade and placed in plastic tubs. Wet paper towels were placed over the plants to prevent desiccation during transport. Plants were transported back to the laboratory where they were cleaned of epiphytes and grazers. Additionally, any intact sediment was washed from roots and rhizome using natural filtered seawater of salinity 27 psu. The whole plant leaves were then separated at the horizontal creeping rhizome, and freezed at −80°C for later use for total phenolics estimation and protein extraction. All the reagents used in this study (otherwise stated) were purchased from Sigma-Aldrich.

### Total phenolic compound estimation

Total content of phenolic compounds was determined spectrophotometrically using Folin-Ciocalteu reagent following Kumar et al. (2011). In brief, methanolic extract (0.4 mL) obtained from 1 g fresh tissue was mixed with 0.15 mL of Folin-Ciocalteu's reagent. After 10 min incubation, 0.45 mL of 20% sodium carbonate solution was added, and the mixture was mixed thoroughly and allowed to stand at room temperature in the dark for 1 h. Absorbance was measured at 725 nm, and total content of phenolic compounds was calculated based on a standard curve of phloroglucinol.

### Protein extraction

**Figure 1:**
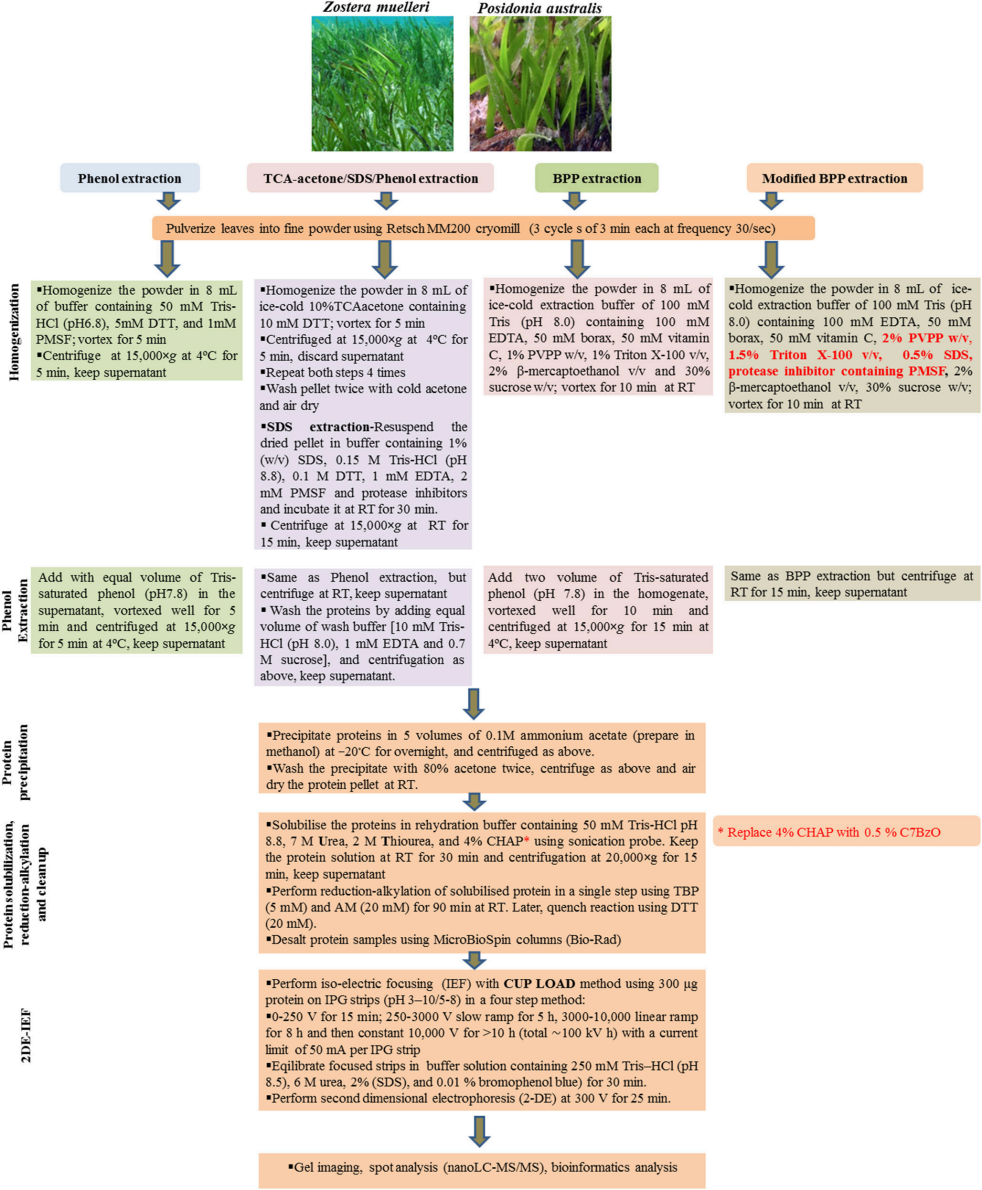
A schematic workflow of all the four tested protein extraction methods including modified BPP in seagrasses *Zostera muelleri* and *Posidonia australis*.

Three commonly used protein extraction methods, phenol extraction, TCA/acetone/SDS-phenol extraction and borax/polyvinyl-polypyrrolidone/phenol extraction were evaluated using seagrass leaves (Figure 1). For each method, whole leaves of both *Z. muelleri and P. australis* (2 g FW) were pulverized using Retsch MM200 cryomill with a 1 cm stainless steel ball for 9 min in three cycles of 3 min each at a frequency of 30/s. Later, the obtained fine talcum-like powder was used for protein extraction.

### Phenol (P) method

The powder was homogenized in 8 mL of buffer containing 50 mM Tris-HCl (pH6.8), 5 mM DTT, and 1 mM PMSF, and vortexed for 5 min. The homogenate was transferred into 2 mL eppendorf tubes and centrifuged at 15,000×g and 4°C for 5 min. The supernatant (crude extract) was transferred to new tubes. Equal volumes of Tris-saturated phenol (pH 8.0) were added to the tubes and phenol extraction was performed using a methodology adapted from Wu et al. (2014a). The mixtures were thoroughly vortexed for 5 min and centrifuged as above for phase separation. The organic phase was transferred in new eppendorf tubes and precipitated using 5 volumes of 0.1M ammonium acetate in methanol at −20°C overnight, and centrifuged as above. The precipitate was washed twice with 80% cold acetone. For each wash, 3 ml of 80% cold acetone was added, and the precipitate was resuspended thoroughly by vortexing and centrifuged as above.

### Trichloroacetic acid/acetone/SDS/phenol (TASP) method

This methodology was adapted from Wu et al. (2014b). The powder was resuspended in 8 mL of ice-cold 10%TCA/acetone containing 10 mM DTT and vortexed for 5 min. The homogenate was then transferred to 2 mL Eppendorf tubes and centrifuged at 15,000×g at 4°C for 5 min. This step was repeated for four times, while re-suspending the pellet in cold TCA/acetone by pipetting and vortexing or drawing in and out of pellet with a 1.0 mL pipette. Finally, the precipitate was washed twice with cold acetone as described above in the phenol extraction. The pellet was collected and dried in fume hood to ensure that all of the acetone has evaporated. The dried pellet was resuspend in sodium dodecyl sulfate (SDS) extraction buffer [containing 1% (w/v) SDS, 0.15 M Tris-HCl (pH 8.8), 0.1 M DTT, 1 mM EDTA, 2 mM PMSF and protease inhibitors cocktail (Roche, Germany)] and incubated at room temperature (RT) for 30 min followed by centrifugation at 15,000 × g at RT for 15 min. The resultant supernatant was transferred into new 2 mL Eppendorf tubes and an equal volume of Tris-saturated phenol (pH7.8) was added, vortexed well for 5 min and centrifuged at 15,000 × g for 5 min at RT. The phenol phase (lower phase) containing the proteins was collected in new eppendorf tubes and mixed well after adding an equal volume of wash buffer [10 mM Tris-HCl (pH 8.0), 1 mM EDTA and 0.7 M sucrose], followed by centrifugation as above. Later, the organic phase was collected in new 2 mL Eppendorf tubes and proteins were precipitated using 5 volumes of 0.1M ammonium acetate in methanol and washed as described above in “phenol extraction.”

### Borax/polyvinyl-polypyrrolidone/phenol (BPP) method

This methodology was adapted from Wang et al. (2007). The powder was resuspended in 8 mL ice-cold extraction buffer consisting of 100 mM Tris (pH 8.0), 100 mM EDTA, 50 mM borax, 50 mM ascorbic acid, 1% PVPP w/v, 1% Triton X-100 v/v, 2% β-mercaptoethanol v/v, and 30% sucrose w/v. After the sample was vortexed for 10 min at room temperature, two volumes of Tris-saturated Phenol (pH 8.0) were added and the mixture further vortexed for 10 min. After centrifugation at 15,000 × g for 15 min at 4°C, the upper phase was transferred to a new centrifuge tube. An equal volume of extraction buffer was added into the new tube, the mixture was then vortexed for 10 min, followed by centrifugation as above. The upper phase was then transferred to a new centrifuge tube and proteins were precipitated using 5 volumes of 0.1M ammonium acetate in methanol and washed as described above in “phenol extraction.”

### Modified borax/polyvinyl-polypyrrolidone/phenol (M-BPP) method

This methodology was adapted from Wang et al. (2007). The powder (obtained by pulverizing the tissue in cryomill) was resuspended in 8 mL of ice-cold BPP extraction buffer as mentioned in the BPP method. However, the extraction buffer was slightly modified by additional incorporation of *the ionic detergent-SDS (0.5% w/v) together with the non-ionic detergent 1.5% triton X-100 (v/v), 2% PVPP (w/v), and protease inhibitor cocktail* (Roche, Germany). The reagents SDS and PVPP were added in the extraction buffer from their stock solutions − SDS (20% w/v) and PVPP (10% w/v) respectively. Protease inhibitor cocktail available commercially in the tablet form was dissolved in the extraction buffer (1 tablet/50 mL extraction buffer) using sonicator water bath. After the sample was vortexed for 10 min at room temperature, two volumes of Tris-saturated Phenol (pH 8.0) were added and then the mixture was further vortexed for 10 min. After centrifugation at 15,000×g for 15 min at 4°C, the upper phase was transferred to a new centrifuge tube. Equal volume of extraction buffer was added into the new tube, the mixture was then vortexed for 10 min, followed by centrifugation as above. The upper phase was then transferred to a new centrifuge tube and proteins were precipitated using 5 volumes of 0.1M ammonium acetate in methanol and washed as described above in “phenol extraction.”

### Protein solubilization, alkylation-reduction, and quantification

The precipitated proteins were solubilized in rehydration buffer containing 50 mM Tris-HCl pH 8.8, 7 M Urea, 2 M Thiourea and 4% CHAPS. *However, for the M-BPP method we replaced CHAPS with 0.5% C7BzO (UTC7)*. Solubilization was followed by the reduction and alkylation of disulfide bonds in a single step using tributylphosphine (reducing agent, 5 mM) and acrylamide monomers (alkylating agent, 20 mM) for 90 min at RT. The reaction was quenched using dithiothreitol (DTT, 20 mM). Protein samples were desalted using MicroBioSpin columns (Bio-Rad) equilibrated with UTC7 according to manufacturer's instructions, followed by centrifugation at 15,000 × g for 10 min. The supernatant was collected for protein quantification and subsequent 2-DE analysis. Protein concentration was determined by SDS-PAGE and densitometry using bovine serum albumin as a standard.

### Two-dimensional electrophoresis (2-DE), gel scanning, and image analysis

Protein (300 μg) was separated by isoelectric focusing (IEF) using a cup-loading method according to Kumar et al. (2017). Immobilized pH gradient (IPG) strips (Bio-Rad, pH 3–10 or 5–8, 11 cm) were passively rehydrated in UTC7 rehydration solution for a minimum of 6 h at room temperature. Isoelectric focusing was conducted in four steps as follows in a Protean IEF device (Bio-Rad): 250 V rapid ramp for 15 min, 4,000 V slow ramp for 8 h, 10,000 V linear ramp for 5 h and then constant 10,000 V for >10 h (total ~100 kV h) with a current limit of 50 mA per IPG strip. The focused strips were then equilibrated in equilibration solution (containing 250 mM Tris–HCl (pH 8.5), 6 M urea, 2% (SDS), and 0.01 % bromophenol blue) for 30 min. Upon equilibration, the strips were directly applied onto a precast 4–20% polyacrylamide gel (Criterion™ IEF Precast Gels, Bio-Rad) for second dimension electrophoresis at constant voltage of 300 V, for 25 min. Gels were then fixed with 40% methanol, 10% acetic acid for 30 min before being stained with Coomassie Stain G-250 and scanned at 600 dots per inch with fluorescence scanner (Typhoon FLA-3500), then analyzed using PDQuest 2-D analysis software, version 8.0 (Bio-Rad, USA). Molecular masses were estimated using a broad-range standard (Precision Plus, Bio-Rad) co-migrating in the SDS-PAGE.

### Protein identification and bioinformatics analysis

Randomly selected protein spots were excised from gels, trypsin digested, and analyzed by LC/MS/MS according to Kumar et al. (2017). Using an autosampler, connected to a nanoLC system (Tempo Eksigent, USA), 10 μL of the sample was loaded at 20 μL/min with MS loading solvent (2% Acetonitrile + 0.2% Trifluoroacetic Acid) onto a C8 trap column (CapTrap. Michrom Biosciences, USA). After washing the trap for 3 min, the peptides were washed off the trap at 300 nL/min onto a PicoFrit column (75 μm × 100 mm) packed with Magic C18AQ resin (Michrom Biosciences, USA). Peptides were eluted from the column and into the source of a QSTAR Elite hybrid Quadrupole-Time-of-Flight mass spectrometer (Applied Biosystems/MDS Sciex) using the following program: 5–50% MS solvent B (98% Acetonitrile + 0.2% Formic Acid) over 8 min, 50–80% MS buffer B over 5 min, 80% MS buffer B for 2 min, 80–5% for 3 min. MS solvent A consisted of 2% Acetonitrile + 0.2% Formic Acid. The eluting peptides were ionized with a 75 μm ID emitter tip that tapered to 15 μm (New Objective) at 2,300 V. An Intelligent Data Acquisition (IDA) experiment was performed, with a mass range of 375–1,500 Da continuously scanned for peptides of charge state 2+ to 5+ with an intensity of more than 30 counts/s. Selected peptides were fragmented and the product ion fragment masses measured over a mass range of 100–1,500 Da. The mass of the precursor peptide was then excluded for 15 s.

Peptides were identified and protein identity inferred using both Mascot (Daemon, v2.4) and PEAKS Studio software (Peaks Studio 8.1, Bioinformatics Solutions Inc., Waterloo, ON, Canada). The settings used were as follows—Fixed Modifications: none; Variable Modifications: deamidation, propionamide, oxidized methionine; Enzyme: semi-trypsin; Number of Allowed Missed Cleavages: 3; Peptide Mass Tolerance: 100 ppm; MS/MS Mass Tolerance: 0.2 Da; Charge State: 2+, 3+, and 4+ (Kumar et al., 2017).

The results of the search were then filtered by including only protein hits with at least one unique peptide and excluding peptide hits with a *p* > 0.05. Peptides were further validated by manual inspection of the MS/MS spectra for the peptide to ensure the b- and y-ion series were sufficiently extensive for an accurate identification. For further protein identification, the Uniprot database of *Z. marina* and the customized database generated by converting ESTs of different seagrasses into protein sequences, were searched using PEAKS Studio v8.1 using the same parameters as Mascot. Later, the PEAKS studio search results were exported into a mzXML file and normalized and quantified using Scaffold Version 4.0 software. The threshold selection for the protein sequences was a PEAKS protein score >20 (the sum of the supporting peptide scores for each distinct sequence that are a representation of the *p*-value in PEAKS as a proxy of the LDF score, which measures the quality of the peptide-spectrum match; Kumar et al., 2017). Only proteins showing at least one peptide with an individual score confidence >20 in PEAKS, when the scaffold parameter was set at a protein threshold of 90% and peptide threshold of 95%, were considered as valid candidates. For these proteins, MS/MS spectra were also manually validated by the presence of a series of at least four *y*-ions.

After PEAKS identification, protein sequences were analyzed using BLAST-P to determine similarity with known proteins in the NCBI database. The threshold was set to a minimal significance of 1e^−3^ and an identity percentage of >25%. The theoretical p*I* and molecular weight of the blast hit was calculated using the ExPASy tool (http://web.expasy.org/compute_pi/). The identified proteins were further annotated using InterproScan (Finn et al., 2017). The Gene Ontology terms were inferred using Interpro2GO (Gene Ontology Consortium, 2015). Subcellular localization of the proteins was assigned using Plant-mLoc (http://www.csbio.sjtu.edu.cn/bioinf/plant-multi/) and manually translated to Gene Ontology terms. GO terms were summarized using the GOSlimViewer tool included in AgBase based on the Plant GOSlim set (McCarthy et al., 2006).

### Western blot analysis

About 2.5 to 25 μg of the isolated proteins were separated via SDS-PAGE and then transferred onto a polyvinylidene difluoride (PVDF) membrane (GE Healthcare) for Western blotting analysis. Western blot analysis was carried out using a 1:500 polyclonal antibody raised against the evolutionarily conserved sequence of Arabidopsis thaliana PEPC purchased from Agrisera, Sweden (1:2,000 dilution) as primary antibody and a goat anti-rabbit IgG-labeled with horseradish peroxidase (HRP) as the secondary antibody. The detection of the immuno-complexes was performed by the Clarity ECL Substrate (Bio-Rad, Australia).

### Statistical analysis

The statistical results were presented as means ± *SD* (standard deviation) of three biological replicates. Statistical analysis, one-way ANOVA, tests was performed with 5% level of significant using the SPSS software (version 12.0).

## Results

Among the four tested protein extraction methods (P, TASP, BPP, and M-BPP), a modified BPP method (M-BPP) with the incorporation of 0.5% SDS, 1.5% Triton X-100, 2% PVPP, and a protease inhibitor cocktail for protein extraction and 0.5% of the zwitterionic surfactant C7BzO for protein solubilization, was found to produce most reproducible gels and highest protein yield (0.79 mg/g fresh weight, FW) for *Z. muelleri* (Table 1). The protein extraction and 2D-IEF work flow for all the tested methods is outlined in Figure 1. M-BPP resulted in a dramatically higher number of protein spots (503, using pH 3-10 strip and 814 protein spots using pH 5-8 strip), which was significantly higher when compared to other methods (Table 1). The protein yield and number of protein spots obtained in all the tested methods followed the order: M-BPP>BPP>TASP>P for *Z. muelleri*. In contrast, all the tested methods were equally good in obtaining a high protein yield with high protein spot numbers for *P. australis*. In general, M-BPP resulted in 40% and 15% higher protein spots in *Z. muelleri* and *P. australis*, respectively when compared to original BPP method.

**Table 1:** Protein yield, protein spot numbers on 2DE and extraction process time in the four tested protein extraction methods.

**Method**	**Protein yield (mg/g fw)**		**Spot number (pI 3–10)**		**Time (h)**
	***Z. muelleri***	***P. australis***	***Z. muelleri***	***P. australis***	
P	0.30 ± 0.05	0.93 ± 0.09	183 ± 22	641 ± 33	1
TASP	0.41 ± 0.07	0.80 ± 0.11	255 ± 25	684 ± 23	3
BPP	0.58 ± 0.04	0.90 ± 0.10	360 ± 19	777 ± 28	1.5
M-BPP	0.79 ± 0.08	1.02 ± 0.08	503 ± 18 (814 ± 30)	898 ± 39 (1082 ± 36)	1.5

*Values in () represent protein spots number on pI 5–8*.

**Figure 2:**
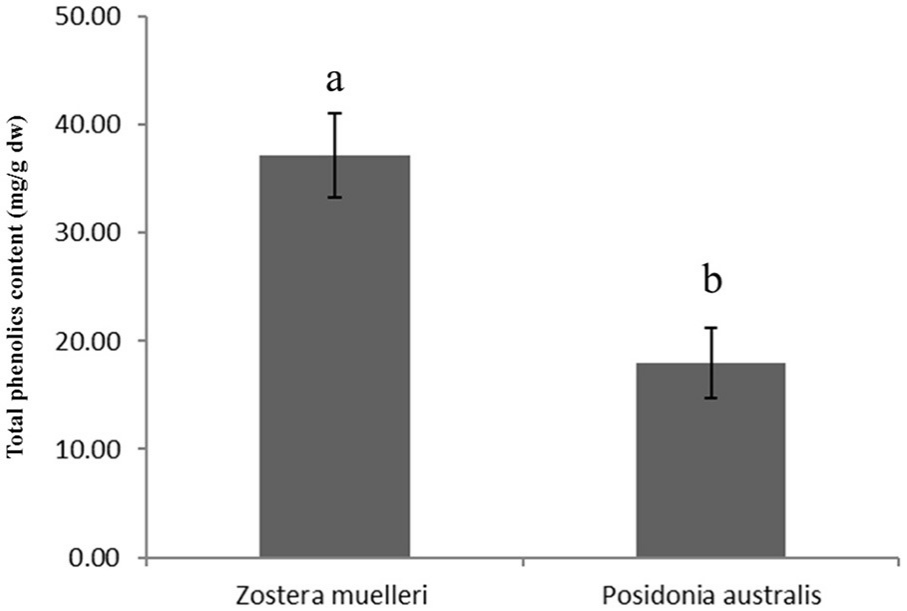
Total phenolics content in the leaves of seagrasses *Zostera muelleri* and *Posidonia australis*. Different lower case letters represent the statistical different at *p* < 0.05.

Distinct qualitative and quantitative differences were noticed in the protein separation pattern between the methods examined in the present study. For example, in both P and TASP methods applied to *Z. muelleri*, the resolved proteins were restricted to a pI range between 5 and 7 and molecular weight of 10–60 KDa with 183 ± 22 and 255 ± 25 protein spots, respectively (Table 1, Supplementary Figure 1). Interestingly, in the TASP method, Rubisco proteins (large subunit) were less abundant when compared to the P method (Supplementary Figures 1A,B) showing an inefficient extraction. Further, proteins within the molecular weight (Mw) ranging from 12 to 20 kDa and pI 4.0–5.25 were more abundant in TASP and BPP extractions than in the P and M-BPP extractions (Supplementary Figure 1B, see the protein spots in red box). Protein identification of these protein spots selected from acidic region of the 2D gels of *Z. muelleri* is provided in Supplementary Table 1. In contrast, for *P. australis* proteins extracted by the P, TASP and BPP methods, most of the proteins were resolved within the Mw 10–100 KDa and pI range 5–8 (Supplementary Figures 2A–C). Interestingly, the M-BPP method extracted a range of proteins that are acidic in nature, pI range between 4 and 5 and Mw 10–80 KDa (Supplementary Figure 2D, see the protein spots marked in red). Protein identification of these protein spots selected from acidic region of the 2D gels of *P. australis* is provided in Supplementary Table 1. Further, in the present study, the quantitative analysis of total polyphenolic compounds in leaf exhibited a significantly higher level (>2-fold) in *Z. muelleri* in contrast to *P. australis* (Figure 2).

**Figure 3:**
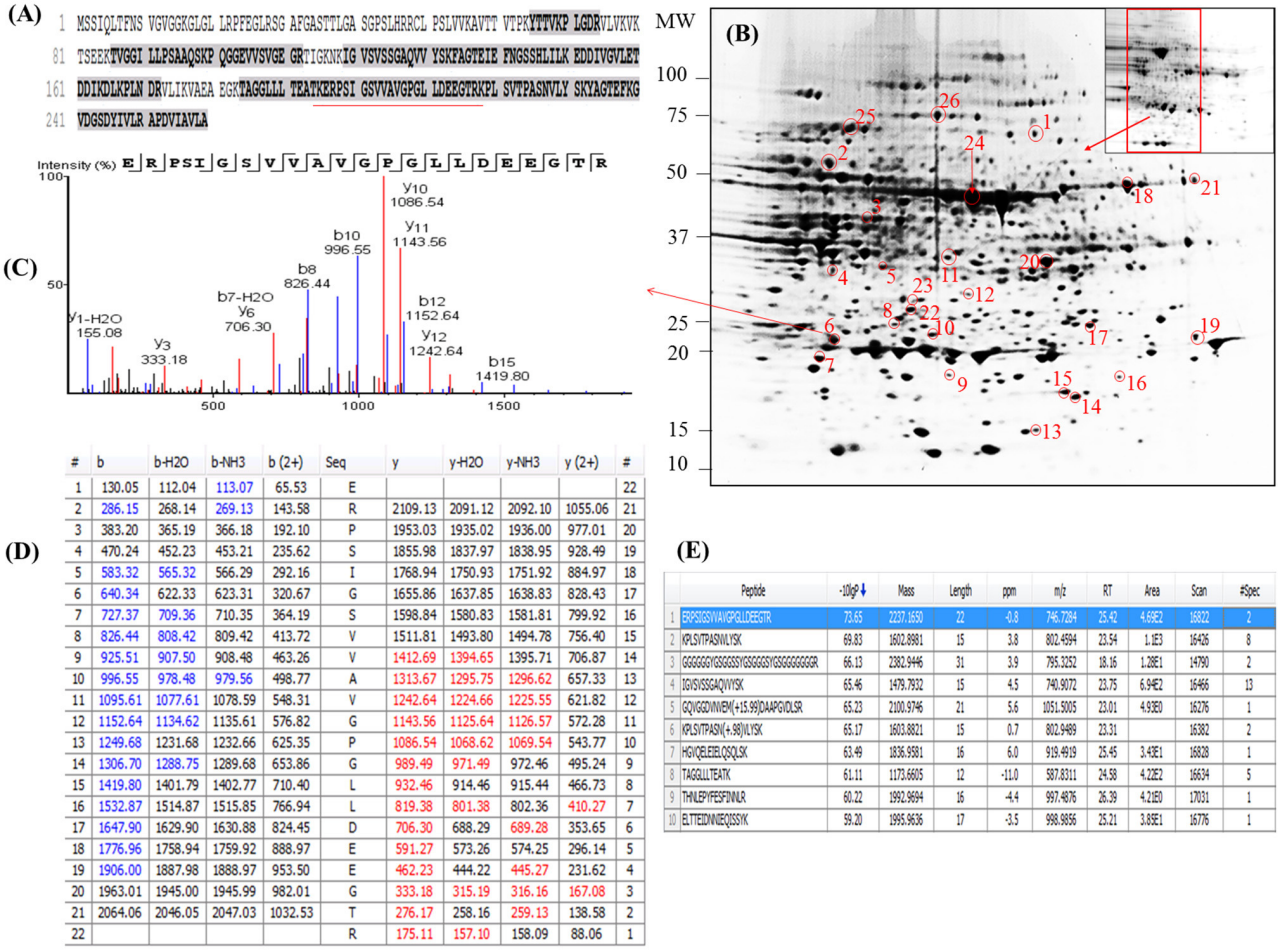
Demonstrative example for the **i**dentification of proteins extracted from leaves of seagrass *Zostera muelleri*. The protein spot (spot 6) was excised, trypsin digested and analyzed using nanoLC-MS/MS. The peptide sequences translated from the cDNA sequences **(A)**, 2D-IEF of proteins resolved on pI range 5–8 **(B)**, annotated peptide mass spectrum **(C)**, ion match summary **(D)**, top 10 annotated peptide with high −10logP score **(E)** are demonstrated for selected spots. Randomly excised protein spots are encircled red and marked with their corresponding numbers. The protein sequences marked with dark letters represent the matched peptides. The matched peptides marked with red under-line was identified and analyzed by nanoLC-MS/MS. The blue and red marked values in ion match summary **(D)** represent the identified and matched amino acids from N- and C-terminal of a peptide sequence.

**Figure 4:**
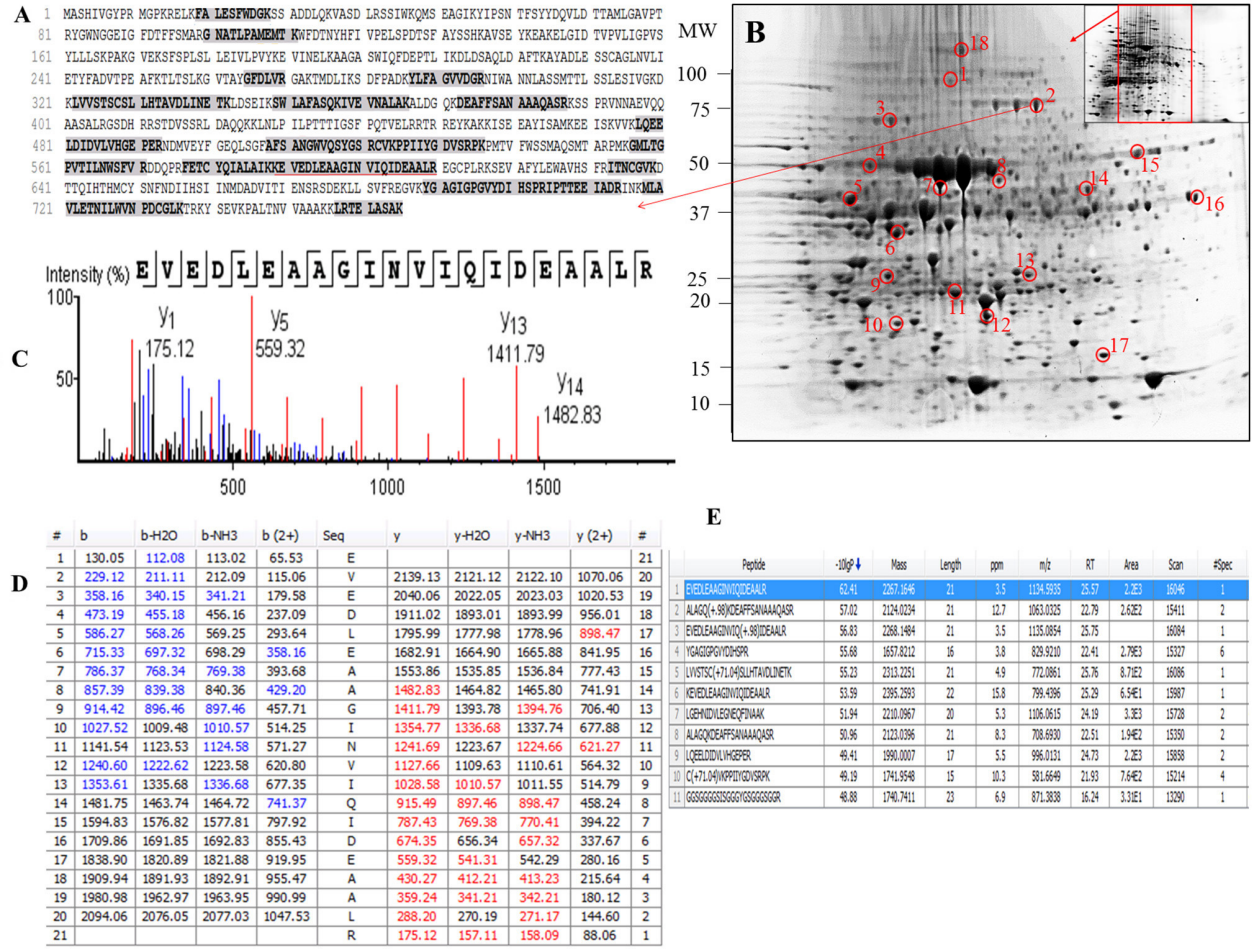
Demonstrative example for the **i**dentification of proteins extracted from leaves of seagrass *Posidonia australis*. The protein spot (spot 2) was excised, trypsin digested and analyzed using nanoLC-MS/MS. The peptide sequences translated from the cDNA sequences **(A)**, 2D-IEF of proteins resolved on pI range 5–8 **(B)**, annotated peptide mass spectrum **(C)**, ion match summary **(D)**, top 10 annotated peptide with high −10logP score **(E)** are demonstrated for selected spots. Randomly excised protein spots are encircled red and marked with their corresponding numbers. The protein sequences marked with dark letters represent the matched peptides. The matched peptides marked with red under-line was identified and analyzed by nanoLC-MS/MS. The blue and red marked values in ion match summary **(D)** represent the identified and matched amino acids from N- and C-terminal of a peptide sequence.

**Figure 5:**
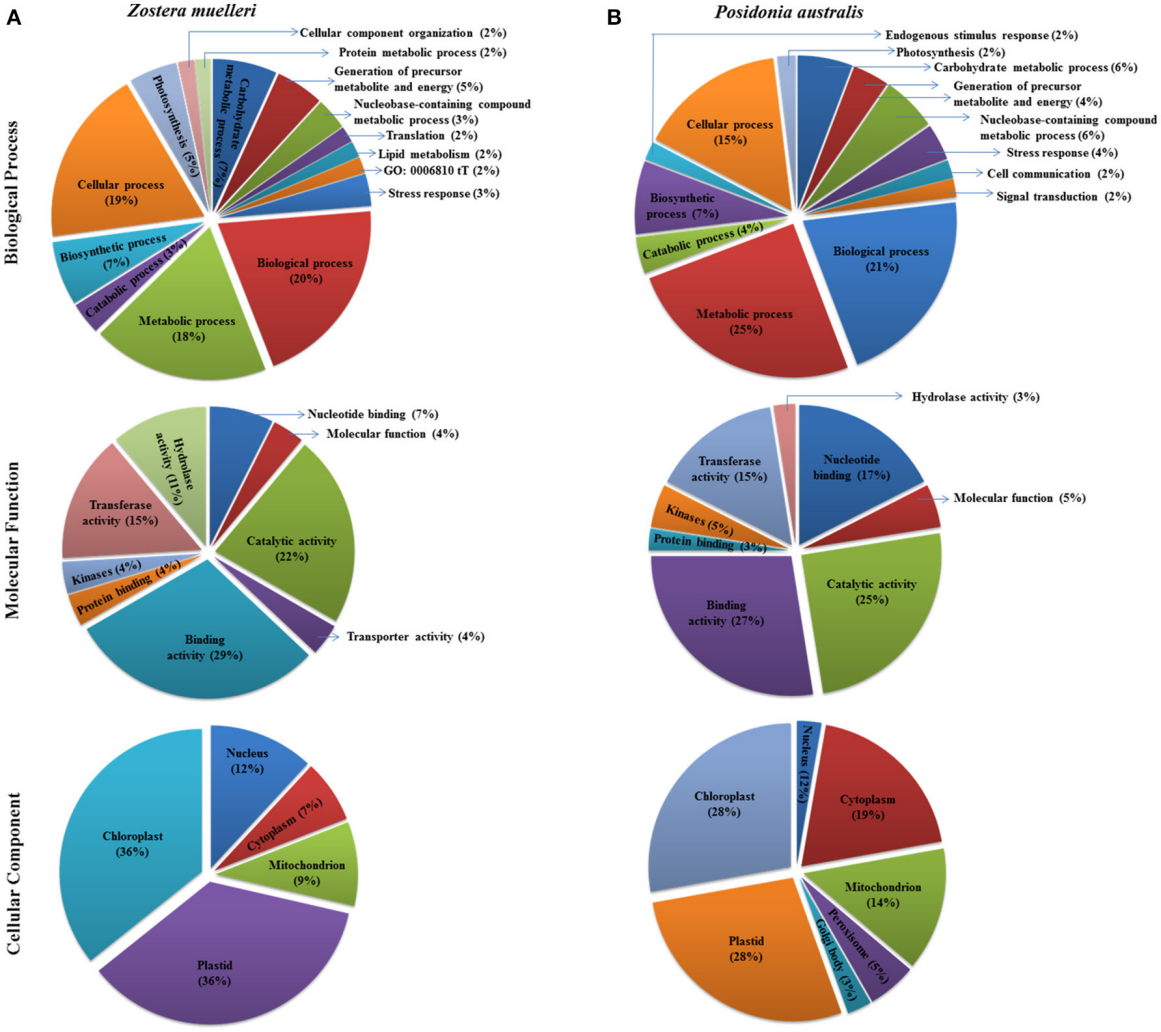
Representation of functional classification of GO annotation distribution for the randomly chosen protein spots (red encircled spots shown in Figures 3, 4) categorized to diverse biological process, molecular function, and cellular component in seagrasses *Zostera muelleri* **(A)** and *Posidonia australis* **(B)**.

**Figure 6:**
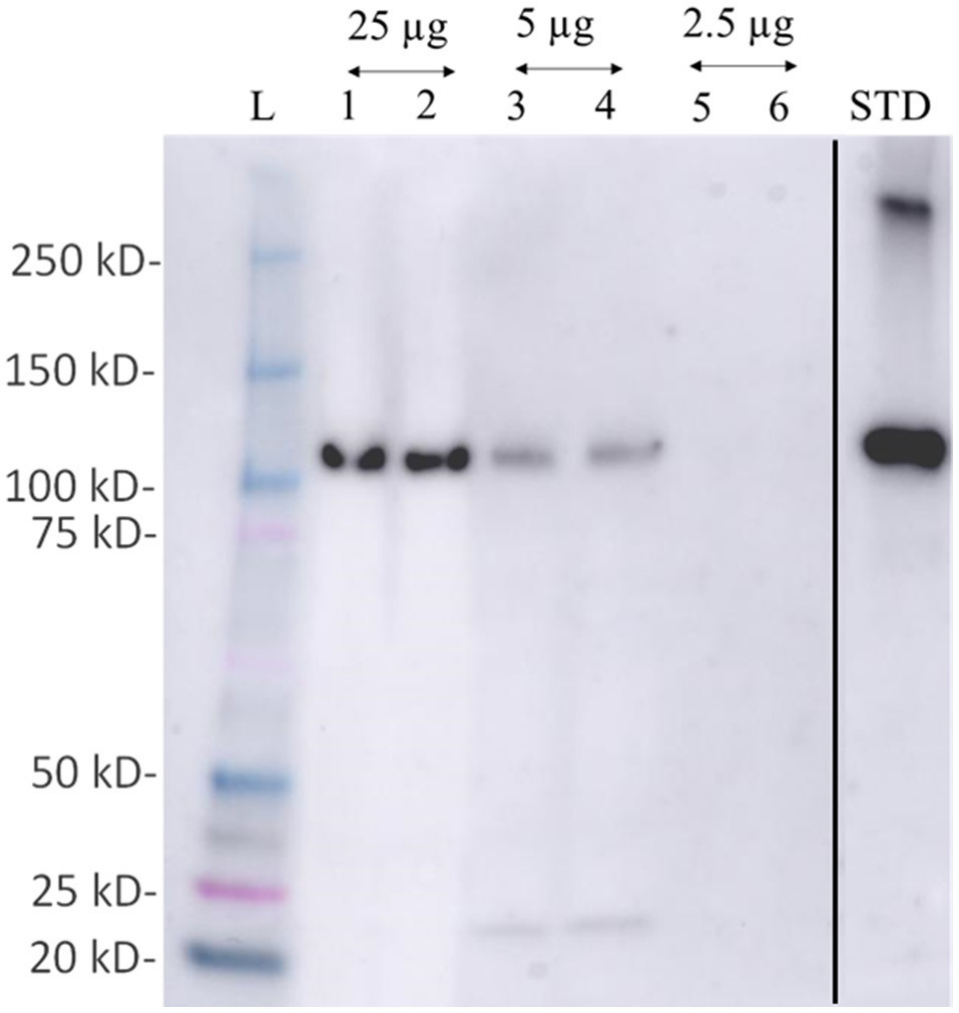
Western blot detection of phosphoenol pyruvate carboxylase (PEPC- a key protein of carbon fixation) protein from total protein extracted using M-BPP method in *Zostera muelleri*. L-molecular weight ladder; lanes 1–6 represent the immunodetection of PEPC using total protein 25 μg (lane 1–2); 5 μg (lane 3–4); and 2.5 μg (lane 5–6) run in duplicates. Std- PEPC standard marker protein. Since, PEPC was not detected in total protein concentration ≤2.5 μg, the respective lanes have been deleted (black line) from the original figure that were run before the Std-PEPC marker lane.

Examination of the 2D-PAGE results for proteins extracted from whole leaves, the modified BPP protocol generated from several hundred (for *Z. muelleri*) to nearly thousand (for *P. australis*) protein spots with a broad distribution in both the horizontal and vertical separation dimensions, within the pI range between 3 and 10 (Supplementary Figures 1, 2), and pI 5–8 (Figures 3B, 4B) and the molecular weight range from 10 to >100 KDa. The spots showed superior resolution with clear background and minimal streaking while the spot shape appeared round or elliptical, even at both cathode and anode extremes (or around high abundant protein regions pI 5–7). To further evaluate the compatibility of this method with MS, protein spots were randomly excised from width and breadth of 2D-IEF gels for nano-LC-MS/MS analysis from both *Z. muelleri and P. australis*. All the selected protein spots indicated by circles and marked with numbers (Figures 3B, 4B) were successfully analyzed by nano-LCMS/MS, identified, and listed in Tables 2, 3. Among the analyzed proteins, spot 6 for *Z. muelleri* (Figure 3B) and spot 2 for *P. australis* (Figure 4B) were randomly chosen to demonstrate their identification in detail. The peptide sequences translated from the cDNA sequences (Figures 3, 4A), annotated peptide mass spectrum (Figures 3, 4C), ion match summary (Figures 3, 4D), annotated top 10 peptide match description (Figures 3, 4E) were demonstrated for selected spots in both seagrasses. The functional classification of these selected proteins belonging to diverse biological, metabolic and cellular processes from both the seagrasses is presented in Figure 5. Finally, we successfully immunoblotted the phosphoenolpyruvate carboxylase (PEPC—a key enzyme in carbon metabolism) in the whole protein of *Z. muelleri* extracted using M-BPP method. We could detect this protein efficiently while using a minimum of 5 μg of total protein (Figure 6).

**Table 2:** Identification of proteins using software PEAKS studio ver 8.0 analyzed by LC-MS/MS for *Zostera muelleri*.

**Spot no**	**Protein name**	**Accession**	**Organism**	**SL^a^**	**pI**		**Mr**		**Score**	**Pep^b^**	**Uni^c^**	**SC**
					**Obs**.	**Thr**.	**Obs**.	**Thr**.				
1	Heat shock protein STI1	gb|KMZ64384.1	*Z. marina*	N	6.78	5.79	75.69	65.94	192.68	17	17	35
2	Inositol-3-phosphate synthase	gb|KMZ57169.1	*Z. marina*	CY	5.72	5.84	64.78	64.95	169.57	14	7	28
3	Phosphoglycerate kinase	gb|KMZ64101.1	*Z. marina*	CL	6.12	8.30	45.68	50.32	205.37	33	26	77
4	Isoflavone reductase-like protein	gb|KMZ72723.1	*Z. marina*	CY	5.73	5.13	35.69	35.23	103.15	7	7	25
5	Enoyl-ACP Reductase	gb|KMZ61785.1	*Z. marina*	CL	6.09	8.69	36.28	39.88	161.88	12	4	40
6	20 kDa chaperonin	gb|KMZ69941.1	*Z. marina*	CL	5.88	8.49	24.87	27.09	213.37	22	20	64
7	ATP synthase delta-subunit	gb|KMZ66253.1	*Z. marina*	M	5.77	9.37	21.92	28.13	182.27	15	6	81
8	Protein thf1	gb|KMZ63330.1	*Z. marina*	CL	6.16	8.87	26.23	33.20	154.37	9	7	41
9	Glutathione S-transferase F9, Phi class	gb|KMZ60880.1	*Z. marina*	CY	6.40	5.46	19.14	23.92	147.20	14	14	63
10	Ribosome-recycling factor	gb|KMZ64562.1	*Z. marina*	CL	6.32	9.16	26.02	30.96	108.74	5	5	32
11	Quinone oxidoreductase-like protein	gb|KMZ66757.1	*Z. marina*	CL	6.38	7.66	35.83	37.99	162.25	11	11	49
12	Phage shock protein A, PspA	gb|KMZ69686.1	*Z. marina*	CL	6.47	8.99	30.14	39.93	150.97	13	13	49
13	hypothetical protein	gb|KMZ71315.1	*Z. marina*	CL	6.81	9.22	15.96	15.25	64.81	2	2	15
14	PsbP-like protein 1	gb|KMZ62962.1	*Z. marina*	CL	6.98	9.22	17.86	28.18	168.68	11	11	66
15	Adenine nucleotide alpha hydrolases like	gb|KMZ58089.1	*Z. marina*	CL/N	6.93	5.63	18.16	18.38	137.32	8	8	57
16	Nascent polypeptide-associated complex, β	gb|KMZ60575.1	*Z. marina*	N	7.18	7.92	19.05	16.35	106.41	6	6	51
17	ATP synthase subunit O, mitochondrial	gb|KMZ64579.1	*Z. marina*	M	7.05	9.08	26.02	30.50	128.56	6	6	32
18	Protein plastid transcriptionally active 16	gb|KMZ73091.1	*Z. marina*	N	7.22	8.98	53.86	53.98	215.51	30	6	60
19	OEE-PsbP	gb|KMZ57551.1	*Z. marina*	M	7.53	8.76	23.18	27.68	200.20	13	11	36
20	Malate dehydrogenase	gb|KMZ65591.1	*Z. marina*	M	6.85	8.23	35.59	36.61	210.24	22	6	67
21	Glycine hydroxymethyltransferase	gb|KMZ69888.1	*Z. marina*	M	7.51	8.79	55.52	57.82	185.66	24	24	62
22	Plasma membrane associated cation-binding protein 1	gb|KMZ61692.1	*Z. marina*	CL	6.23	5.59	28.02	21.60	142.10	14	14	64
23	Proteasome subunit alpha type-6	XP_010937324.1	*Z. marina*	N	6.25	6.00	29.37	27.42	110.29	9	5	49
24	RuBisCo, large subunit, partial	gb|AIZ98377.1	*Z. angustifolia*	CL	6.51	6.09	48.84	50.21	209.26	24	0	56
25	Transketolase	gb|KMZ75731.1	*Z. marina*	CL	5.94	5.93	78.87	81.03	282.02	43	17	69
26	Methionine synthase	gb|KMZ76082.1	*Z. marina*	CL	6.32	5.92	84.59	84.67	306.50	34	10	53

a Subcellular location of proteins was predicted using the online Plant-mPLoc server (http://www.csbio.sjtu.edu.cn/ bioinf/ plant-multi);

b Exclusive unique peptide count;

c *Exclusive unique spectrum count; SC, sequence coverage; obs, observed; theo, theoretical; pI, isoelectric point; Mr, molecular weight; CL, chloroplast; CY, cytoplasm, M, mitochondria; N, nucleus; methionine synthase, 5-methyltetrahydropteroyltriglutamate-homocysteine S-methyltransferase; OEE-Oxygen evolving enhancer protein*.

**Table 3:** Identification of proteins using software PEAKS studio ver 8.0 analyzed by LC-MS/MS for *Posidonia australis*.

**Spot no**	**Protein name**	**Accession**	**Organism**	**SL^a^**	**pI**		**Mr**		**Score**	**Pep^b^**	**Uni^c^**	**SC**
					**Obs**.	**Thr**.	**Obs**.	**Thr**.				
1	Aconitate hydratase	gb|KMZ63807.1	*Z. marina*	CY/M	6.53	6.48	98.62	108.15	140.5	11	1	12
2	Methionine synthase	gb|KMZ76082.1	*Z. marina*	CL	6.95	5.92	85.86	84.67	185.22	20	1	32
3	Transketolase	gb|KMZ75731.1	*Z. marina*	CL	6.23	5.93	74.17	81.02	115.73	5	1	9
4	Adenosylhomocysteinase	gb|KMZ66813.1	*Z. marina*	PX	6.14	5.60	49.83	53.56	128.50	15	7	21
5	Phosphoglycerate kinase	gb|KMZ58914.1	*Z. marina*	CL	6.09	6.21	41.22	42.66	268.72	33	8	71
6	Ferredoxin–NADP reductase	gb|KMZ70342.1	*Z. marina*	CL	6.26	8.68	34.15	40.56	182.21	20	15	52
7	GDP-mannose 3,5-epimerase 1	gb|KMZ73857.1	*Z. marina*	GB	6.48	5.57	44.73	42.80	209.27	18	1	45
8	Isocitrate dehydrogenase (NADP(+))	gb|KMZ71727.1	*Z. marina*	CL/CY/M/PX	6.77	5.81	48.32	50.21	149.10	13	4	29
9	Probable ATP synthase 24 kDa subunit,	ref|XP_008791367.1	*P. dactylifera*	M	6.21	7.74	27.82	28.02	68.78	2	2	24
10	Rhodanese-like domain-containing protein 14	ref|XP_002274646.1	*V. vinifera*	CL	6.26	9.00	18.55	25.92	117.24	6	6	33
11	Glutathione S-transferase F7, Phi class	gb|KMZ61632.1	*Z. marina*	CY	6.55	5.44	23.78	24.38	97.74	6	5	25
12	Glutathione Peroxidase	gb|KMZ63257.1	*Z. marina*	CL/M	6.71	6.59	19.20	18.3	114.4	7	4	45
13	Ascorbate peroxidase 4	gb|KMZ62361.1	*Z. marina*	CL	6.91	8.87	27.36	35.10	88.02	3	2	25
14	ATP sulfurylase 1	ref|NP_188929.1	*A. thaliana*	CY	7.18	6.34	44.68	51.45	152.81	11	8	55
15	NADP-dependent G3PDH	ref|XP_002279374.1	*V. vinifera*	CY	7.47	6.67	56.82	53.17	113.39	5	2	60
16	G3PDH	gb|KMZ64911.1	*Z. marina*	CY	7.72	6.97	41.55	36.47	119.45	7	2	23
17	Nucleoside diphosphate kinase 3-like	ref|XP_004244635.1	*S. lycopersicum*	CY/CL/M/N	7.29	9.69	16.25	25.47	88.49	5	5	18
18	RuBisCO, large subunit	gb|AIZ98377.1	*Z. marina*	CL	6.61	6.09	120.18	50.21	109.89	7	0	34

a Subcellular location of proteins was predicted using the online Plant-mPLoc server (http://www.csbio.sjtu.edu.cn/bioinf/plant-multi);

b Exclusive unique peptide count;

c *Exclusive unique spectrum count; SC, sequence coverage; obs, observed; theo, theoretical; pI, isoelectric point; Mr, molecular weight; CL, chloroplast; CY, cytoplasm, M, mitochondria; GB, golgi body; N, nucleus; PX, peroxisome; G3PDH, glyceraldehyde-3-phosphate dehydrogenase; methionine synthase, 5-methyltetrahydropteroyltriglutamate-homocysteine S-methyltransferase; A. thaliana, Arabidopsis thaliana; V. venifera, Vitis Vinifera; P. dactylifera, Phoenix dactylifera; Solanum lycopersicum, S. lycopersicum*.

## Discussion

In marine macrophytes, proteomics-based approaches are still in the incipient stage for use in understanding the acclimation and/or tolerance mechanisms to environmental cues. Marine macrophyte protein extraction is particularly difficult due to low protein concentrations and contaminant co-extraction. Due to this, a specific protein extraction protocol needs to be optimized and established for extracting the maximum amount and range of proteoform species in an unbiased manner, not favoring particular proteins while leaving others behind and insoluble. Toward this, protein extraction protocols have been refined to produce well-resolved 1-D and/or 2-D PAGE images in seagrass *Posidonia oceanica* (Migliore et al., 2007; Spadafora et al., 2008; Mazzuca et al., 2009; Dattolo et al., 2013) and *Cymodocea nodosa* (Piro et al., 2015). These studies utilized the universal protein extraction protocol wherein protein is precipitated in TCA and 2-ME in cold acetone, followed by several rinsing steps with 2-ME in cold acetone and finally, resuspension of the dry pellet in a buffer optimized for IPG strips. Considering their positive results, we tested phenol (Wu et al., 2014a) and the universal method (which is based on TCA-acetone-SDS-Phenol extraction; Wu et al., 2014b) for protein extraction in *Z. muelleri*. This method provided poor results on 2D-IEF when applied to *Z. muelleri* leaves (Supplementary Figures 1A,B), however worked well with *P. australis*, suggested that the protocol for protein extraction is plant and species-specific. Subsequently, we explored the BPP method of protein extraction which has been successfully employed in the halophytes *Salicornia europaea* (Wang et al., 2007), *Thellungiella halophile* (Wang et al., 2013), and *Sesuvium portulacastrum* (Yi et al., 2014). The BPP method was comparatively better in terms of higher protein yield and number of visible protein spots for *Z. muelleri* leaf samples resolved on 2D-IEF using 3–10 IPG strips. Therefore, we decided to further modify the BPP method as described to define a protocol better suited to *Z. muelleri*.

Interestingly, the M-BPP method resulted in 40 and 15% higher number of protein spots when compared to the original BPP protein extraction method in *Z. muelleri and P. australis*, respectively. Moreover, the M-BPP method for protein extraction, like the BPP method, was completed in just half of the time it takes for the TASP method, which is a significant outcome considering the protein yield, number of spots and minimal background staining observed on the 2D gels. To the best of our knowledge, this is the first report on extracting the protein from seagrasses using BPP based extraction protocols. However, historically, TCA/acetone/Phenol based protein extraction methods have been employed in the seagrasses *P. oceanica* (Spadafora et al., 2008; Dattolo et al., 2013) and *C. nodosa* (Piro et al., 2015) exposed to abiotic stresses. Spadafora et al. (2008) demonstrated the 2DE based proteome profiling in the juvenile, intermediate and adult leaves in *P. oceanica*, revealing mainly abundant proteins as detectable, while less abundant proteins that could be differentially expressed among the studied tissues were hard to identify. There is only a single report in *P. oceanica* wherein a 2D-PAGE based proteomic approach was employed to address the low light acclimation response with detection of >2,600 protein spots on 2D-PAGE from leaf tissues, however only 26 proteins were shown to be differentially expressed in low-light conditions. Among the differentially regulated proteins, <50% proteins were identified through mass spectrometry analyses, however others could not be identified due to lack of significant genomic and proteomic database for *Posidonia* sp. (Mazzuca et al., 2009). Proteome profiling in seagrasses has subsequently been performed using labeled free 1-DE followed by MALDI-TOF based semi-quantitative analysis to study their stress response to altered light and salinity conditions (Dattolo et al., 2013; Piro et al., 2015).

The lower protein yield obtained with the P and TASP methods when applied to *Z. muelleri* in contrast to the yield from *P. australis* could be due to a less efficient removal of polyphenolic compounds and other interfering substances including complex cell wall polysaccharides and pigments. The coextraction of non-protein cellular components such as phenolics and polysaccharides adversely affects protein migration, resulting in streaking and smearing in 2D-PAGE with a reduction in the number of distinctly resolved protein spots (Wu et al., 2014a). In the present study, the quantitative analysis of total polyphenolic compounds in leaves exhibited a significantly higher level in *Z. muelleri* in contrast to *P. australis*. The low cellular proteins content (due to the presence of large vacuoles) and presence of high content of interfering compounds such as phenolics or complex polysaccharides could be a possible reason for not obtaining high quality 2D gels in *Z. muelleri* with these methods. *Z. muelleri* leaf tissues have been shown to rich in phenolic compounds like coumaric acid, cinnamic acid; flavanoids, as well as lignins, and carbohydrates (Kuzhiumparambil et al., in press). These compounds are known to form not only the hydrogen bonds with proteins but they also form irreversible complexes with proteins by oxidation and covalent condensation which leads to charge heterogeneity resulting in streaking of gels (Wu et al., 2014a). Further, co-extraction of carbohydrates blocks the gel pores causing precipitation and prolonged focusing time, which may also result in loss of protein spots and streaks in the gels (Carpentier et al., 2005). Ferrat et al. (2012) also demonstrated a significantly higher level of phenolics in the leaves of *Z. marina* in contrast to *P. oceanica*. Altogether, this suggest that M-BPP method was efficient in removing the polysaccharides and phenolics compared to P and TASP, thus resulted in 2DE gels with clear background and minimal streaking for *Z. muelleri*.

Further, TASP involves the TCA/acetone based precipitation which typically requires extensive washing until the protein pellet becomes colorless, which is time-consuming and results in the proteins having extended exposure to low pH. This may lead to protein degradation or modifications (Wang X. et al., 2016) as also observed in our present study with low number of protein spots on 2DE gels for *Z. muelleri*. Further, in the TASP method, salt ions should be somewhat eliminated during protein precipitation with TCA, however, the sample produced distinct horizontal streaks specifically for *Z. muelleri* proteins (Supplementary Figure 1B). Moreover, in the TASP method following protein precipitation, protein re-solubilization was difficult, necessitating solubilization using a sonication probe (3 times each with 5 s) while keeping the protein sample cold. Similar to our findings, dissolution of proteins following precipitation was also found to be quite difficult in TCA based methods which could lead to a lower protein yield in different tissues of *Cajanus cajan* (Singh et al., 2015). However, in contrast to the TASP method in present study, the protein pellets obtained in the BPP and M-BPP methodologies were easily dissolved in solubilization buffer (UTC− Urea+Thiourea+ CHAPS/C7BzO) in the present study. C7BzO has previously been shown to solubilize more proteins from plant samples at a lower concentration than CHAPS (Luche et al., 2003).

In present study, the incorporation of an ionic detergent (0.5% SDS) together with a non-ionic detergent (1.5% Triton X-100), 2% PVPP and protease inhibitor cocktail into the extraction buffer of the M-BPP method appeared not only to increase the protein yield but also significantly reduced the interfering substances while inhibiting the protease activity and preventing the protein degradation and phenolics oxidation. The presence of both ionic and non-ionic detergents in the M-BPP protein extraction buffer seemed efficient in breaking lipid-lipid and lipid-protein interactions, thereby isolating membrane proteins such as H+-transporting ATP synthase, plasma membrane intrinsic protein (PIP) aquaporins, and annexins (Vâlcu and Schlink, 2006). Incorporation of water-insoluble PVPP into the extraction buffer, which forms hydrogen bonds with phenolic compounds (1–15%, depending on the content of polyphenolics and pectin content), has been suggested as the most efficient practice for the removal of phenolics from plant extracts (Isaacson et al., 2006). Incorporation of ascorbic acid, borax, PVPP, and β-mercaptoethanol into the protein extraction buffer have been previously suggested to extract storage proteins from the recalcitrant tissues of olive leaf (Garcia et al., 2000), from tropical trees (Tian et al., 2003) and halophytes (Wang et al., 2007, 2013; Yi et al., 2014) possibly by preventing the oxidization of polyphenol to polyquinones and the activity of many oxidizing enzymes under highly reduced conditions of the extraction buffer. Historically, BPP based protein extraction methods has been demonstrated as effective for the subcellular fractionation of laticifer latex in *Hevea brasiliensis* (Wang et al., 2010) and in the starch rich tuberous roots of Casava (*Manihot esculenta*; Wang D. et al., 2016) producing protein extracts compatible with 2-DE and MS.

The protein spots that were randomly excised from width and breadth of 2D-IEF gels obtained from M-BPP method for both *Z. muelleri* and *P. australis*, of protein solubilized in UTC7 prior to IEF were successfully analyzed by nano-LC-MS/MS analysis. Progress in the area of proteomics heavily relies on the development of analytical tools for the sensitive, selective, and high-throughput studies of protein analytes (Aebersold and Goodlett, 2001). MS has evolved into a primary analytical tool for proteomics research, especially when coupled with high resolution separation techniques, due to the high information content that can be derived from these coupled techniques (Aebersold and Mann, 2003). Advances in MS have been substantially facilitated by two ionizations techniques; electrospray ionization (ESI) and matrix-assisted laser desorption/ionization (MALDI). ESI-MS produces highly charged ions directly from liquids and is therefore useful for coupling to liquid separations; however MALDI is fast and efficient and has a high tolerance to non-volatile buffers and impurities (Aebersold and Goodlett, 2001). Mass spectrometry enables unambiguous identification of proteins by accurate mass measurements of gas-phase protein and peptide ions and peptide fragment ions. ESI-MS linked to nanoliter-flow HPLC systems are preferred proteomic analytical platform in providing separation, mass determination, and amino acid sequencing in one analytical setup (LC-MS/MS; Aebersold and Mann, 2003). Protein identification using LC-MS/MS is a concentration sensitive technique and an extraction technique that results in a higher intensity spot on 2D-PAGE will result in more peptides of higher abundance once trypsin digested. Thus, higher abundance peptides will produce more comprehensive fragmentation and thus sequence data (Aebersold and Mann, 2003), providing more confidence in the peptide identifications as observed in present study using the M-BPP method. In addition, as peptides of different compositions do not ionize with equal efficiencies, a higher concentration of protein and thus peptides obtained from trypsin digested protein spots in the present study increased the chances of detecting poorly ionizing peptides and lead to greater protein sequence coverage and more confidence in the protein identification. For *Z. muelleri*, all the twenty six excised protein spots were identified and classified into diverse functionally categories wherein the majority of them belong to biological, cellular and metabolic processes with catalytic, binding and transferase activities. For most of the identified proteins the PEAKS score ranged between 103.15 and 306.5 (except spot 13, score 64.81), with a high number of exclusive unique spectrum count (2–26), high number of exclusive peptide count (2–43), and high sequence coverage (15–81%). There were few proteins wherein exclusive unique peptide count and/or exclusive unique spectrum count were low (spot 13, 16, 17, and 24), however their PEAKS score and sequence coverage were significantly higher confirming their identity. Many of these identified proteins have recently been found to be differentially regulated in photo-acclimation responses (Kumar et al., 2017). Specifically, malate dehydrogenase, glycine hydroxymethyl transferase, transketolase, inositol-3-phoaphate sysnthase, glutathione S-transferase, and phosphoglycerate kinase have been suggested to play crucial role in acclimation by providing extra energy requirements and enhancing antioxidants levels during high light induced stress (Kumar et al., 2017). Recently, the functionality of many of the identified proteins in this study has been discussed in other seagrasses species such as *P. oceanica* and *C. nodosa* for their involvement in acclimation/tolerance to low light, salinity and CO_2_ vents (Dattolo et al., 2013; Piro et al., 2015; Olivé et al., 2017; Procaccini et al., 2017). Similar to *Z. muelleri*, the identified proteins in *P. australis* were able to be categorized to diverse biological processes, localized to various cellular components, showing considerably high scores, higher exclusive unique peptide and spectrum counts, and sequence coverage.

Further, the proteins spots that were excised from the acidic region of 2D gel for *Z. muelleri* were successfully analyzed using LCMS/MS and identified as actin, huntingtin-interacting protein K, putative RNA binding protein, 60S acidic ribosomal protein, however others were identified as hypothetical proteins, which is a common occurrence in all organisms and not restricted to plants. However, the proteins that were excised from the acidic region of 2D gel of *P. australis* were identified as profilin-1, ferrodoxin, rubisco activase, 60S acidic ribosome protein, peroxiredoxin, RNA binding protein, ankyrin repeat containing protein, stromal heat shock protein, fructose bi-phosphatase, thiamine thiazole synthase, NADH-ubiquinone oxidoreductase, and ATPase-subunit. Many of these identified proteins specifically ferrodoxin, rubisco activase, actin, NADH-ubiquinone have recently been found to be differentially regulated in photo-acclimation responses (Kumar et al., 2017).

PEAKS analysis results revealed that the proteins spot selected for demonstration purpose in *Z. muelleri* (spot 6, Figure 3) was a 20 kDa chaperonin and in *P. australis* (spot 2, Figure 4) was 5-methyltetrahydropteroyltriglutamate-homocysteine S-methyltransferase (commonly known as methionine synthase-cobalamin independent) respectively. Recently, the crucial role of chaperonin and methionine synthase in providing tolerance to elevated light exposure in *Z. muelleri* has been evidenced (Kumar et al., 2017). A high quality peptide sequence match, annotated peptide mass spectrum and ion match summary obtained for both these and other randomly selected protein spots using LC/MS/MS suggested that the modified BPP method is compatible with MS and can be used for further proteome mapping of *Z. muelleri* and *P. australis* leaf tissues. BPP based protein extraction, 2D-IEF and subsequent analysis of differentially regulated proteins using MALDI-TOF-MS has been successfully employed in the halophytes *S. europaea* (Wang et al., 2007), *T. halophile* (Wang et al., 2013), and *S. portulacastrum* (Yi et al., 2014).

Finally, we successfully performed western blot analysis using the proteins extracted from M-BPP method for the detection of phosphoenolpyruvate carboxylase (PEPC—a key enzyme in carbon metabolism). Immunoreactive bands detected by the anti-PEPC antibody were reported. As expected, a band at an apparent molecular mass of 110 kDa was recognized. This band lies in the molecular mass range of *A. thaliana* PEPC (105–110 KDa). We could detect this protein efficiently while using a minimum of 5 μg of total protein. Recently, a significant down regulation of the PEPC transcript was observed in *C. nodosa* collected from CO_2_ vent site indicating an overall reduction of metabolic processes related with photosynthesis (Olivé et al., 2017).

## Conclusion

By optimizing and modifying the BPP (M-BPP) method as described in this study, we succeed in obtaining a higher yield of proteins from seagrass (*Z. muelleri and P. australis*) leaves. The obtained proteins from both the seagrasses are resolved into several hundreds to thousands of protein spots on 2D-PAGE. The improved method results in high quality 2D-PAGE spot patterns and immunoblots free from background smearing and streaking, without detrimentally affecting protein identification by LC-MS/MS analysis. This method is rapid and requires only 1.5 h compared to the universal TASP method (3 h), and can be used for routine protein extraction from marine angiosperm plants for proteome mapping. To the best of our knowledge, this is the first time that the BPP method has been examined and modified for its successful implementation for the proteome analysis of marine macrophytes. Further, it is highly likely that the results of proteomic studies when integrated with allied omic platforms, such as transcriptomics or metabolomics, will provide better insights on the acclimation/tolerance mechanisms of seagrasses in response to natural and anthropogenic pressure. This will further deepen our understanding of systems biology and will allow identification of the metabolic pathways that are crucial for the survival of marine macrophytes under future climate change scenarios.

## Author contributions

ZJ, MaK, MP, and PR conceived and designed research. ZJ, MaK, and MPP performed 2D-IEF and protein identification using LC-MS/MS and analyzed the data. PD and MiK performed Western Blot analysis while kindly providing the primary and secondary antibodies, and standard for PEPC enzyme. MaK, ZJ, and MPP, wrote manuscript. TK assisted in bioinformatics analysis. PR revised and edit the manuscript. All authors read and approved the manuscript.

### Conflict of interest statement

The authors declare that the research was conducted in the absence of any commercial or financial relationships that could be construed as a potential conflict of interest.

## Supplementary material

Supplementary Figure 1 2D-IEF analysis of extracted proteins from the leaf tissue of seagrass *Zostera muelleri* among the four protein extraction methods– phenol, P **(A)**; TCA/acetone/SDA/Phenol, TASP **(B)**; borax/polyvinyl pyrrolidone/phenol, BPP **(C)**; and modified BPP, M-BPP **(D)** extraction, respectively, on linear gel strip pH 3–10. Randomly excised protein spots are encircled and marked with their corresponding numbers. The red boxes indicated the regions wherein few acidic protein spots were randomly excised and analyzed by nanoLC-MS-MS. The green circle represent the RuBisCO large subunit protein identified and reported by Kumar et al. (2017).

Supplementary Figure 2 2D-IEF analysis of extracted proteins from the leaf tissue of seagrass *Posidonia australis* among the four protein extraction methods– phenol, P **(A)**; TCA/acetone/SDA/Phenol, TASP **(B)**; borax/polyvinyl pyrrolidone/phenol, BPP **(C)**; and modified BPP, M-BPP **(D)** extraction, respectively, on linear gel strip pH 3–10. Randomly excised protein spots are encircled and marked with their corresponding numbers. The red boxes indicated the regions wherein few acidic protein spots were randomly excised and analyzed by nanoLC-MS-MS.

Supplementary Table 1 Identification of proteins using software PEAKS studio ver 8.0 from the excised spots resolved on 2DE (Listed in Tables 1, 2; and red encircled spots shown in Supplementary Figures 1, 2), analyzed by LC-MS/MS for seagrasses *Zostera muelleri* and *Posidonia australis*.
