# Supplementary figures and images for: Development of an Efficient Protein Extraction Method Compatible with LC-MS/MS for Proteome Mapping in Two Australian Seagrasses Zostera muelleri and Posidonia australis
Source: Front Plant Sci. 2017 Aug 15;8:1416. doi: 10.3389/fpls.2017.01416 (PMC5559503; doi:10.3389/fpls.2017.01416)

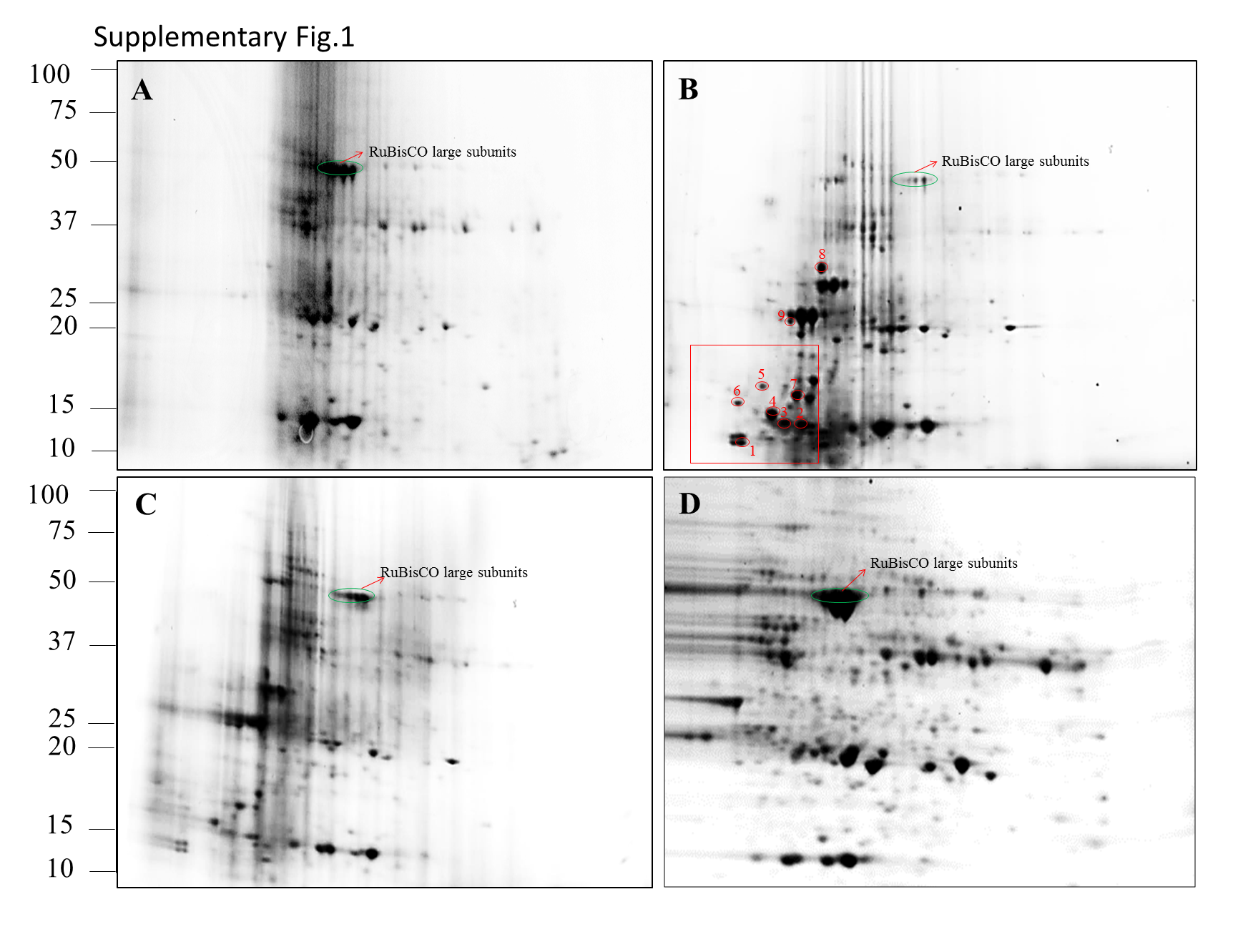

Supplement: Supplementary Figure 1 — 2D-IEF analysis of extracted proteins from the leaf tissue of seagrass Zostera muelleri among the four protein extraction methods– phenol, P (A); TCA/acetone/SDA/Phenol, TASP (B); borax/polyvinyl pyrrolidone/phenol, BPP (C); and modified BPP, M-BPP (D) extraction, respectively, on linear gel strip pH 3–10. Randomly excised protein spots are encircled and marked with their corresponding numbers. The red boxes indicated the regions wherein few acidic protein spots were randomly excised and analyzed by nanoLC-MS-MS. The green circle represent the RuBisCO large subunit protein identified and reported by Kumar et al. (2017). [file Image1.TIF]

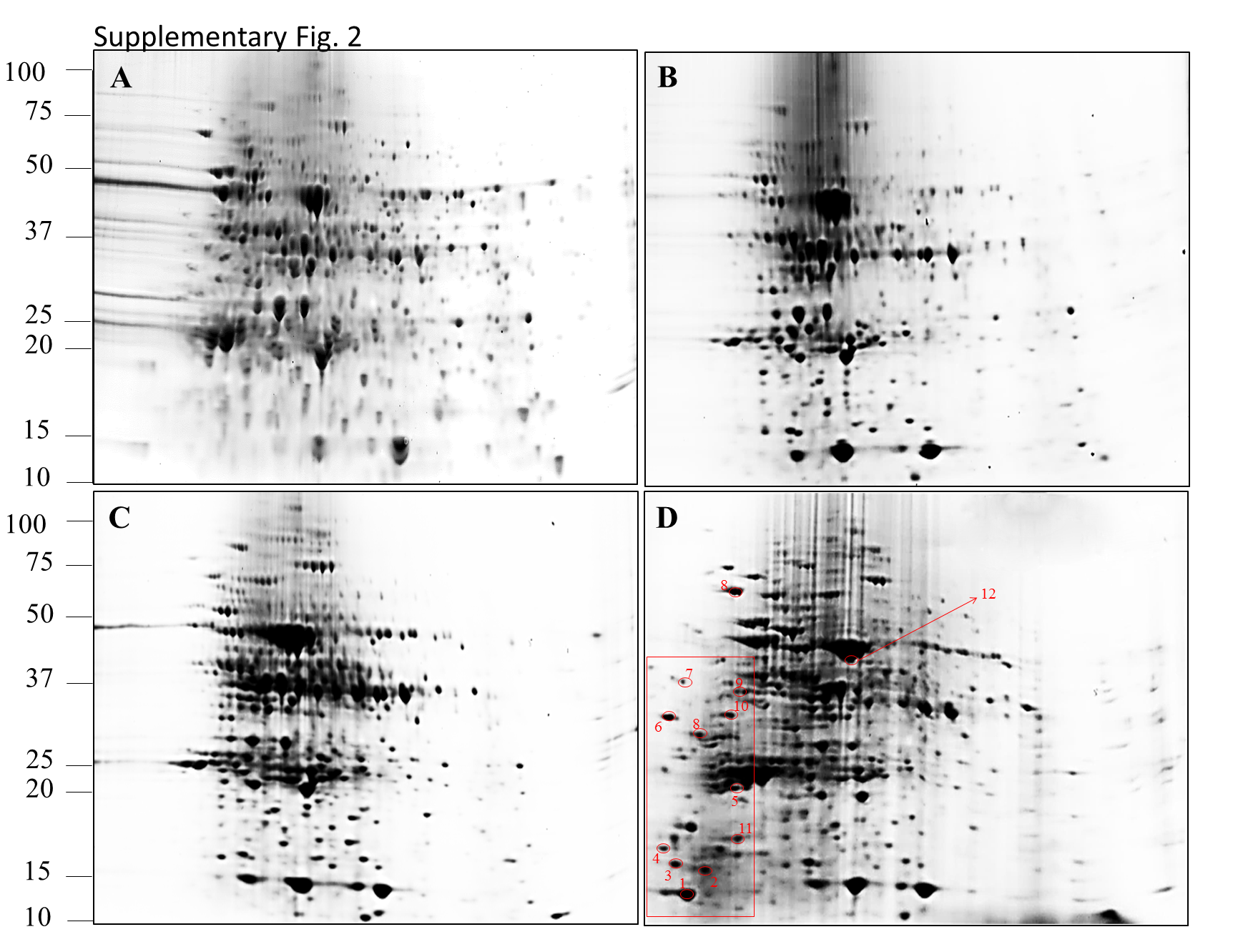

Supplement: Supplementary Figure 2 — 2D-IEF analysis of extracted proteins from the leaf tissue of seagrass Posidonia australis among the four protein extraction methods– phenol, P (A); TCA/acetone/SDA/Phenol, TASP (B); borax/polyvinyl pyrrolidone/phenol, BPP (C); and modified BPP, M-BPP (D) extraction, respectively, on linear gel strip pH 3–10. Randomly excised protein spots are encircled and marked with their corresponding numbers. The red boxes indicated the regions wherein few acidic protein spots were randomly excised and analyzed by nanoLC-MS-MS. [file Image2.TIF]
